# Evaluation of the Quantitative Myasthenia Gravis Score and Grip Strength in Chinese Patients With Myasthenia Gravis: An Observational Study

**DOI:** 10.3389/fneur.2021.782980

**Published:** 2021-12-24

**Authors:** Jinghao Li, Senhui Weng, Sen Lin, Linwen Huang, Xiaojun Yang, Bo Liang, Jiaxin Lu, Qilong Jiang

**Affiliations:** First Affiliated Hospital of Guangzhou University of Chinese Medicine, Guangzhou, China

**Keywords:** myasthenia gravis, quantitative myasthenia gravis score, grip strength, scale, items

## Abstract

**Introduction:** The quantitative myasthenia gravis score is a commonly used scale for evaluating muscle weakness associated with myasthenia gravis (MG). It has been reported that some items used in the scale have low discriminative properties. However, there has been no research investigating the applicability of the quantitative MG score (QMGS) in Chinese patients with MG. In addition, the scoring method and ranges of grip strength items in QMGS need to be further evaluated.

**Methods:** This study included 106 Chinese patients with MG, enrolled between September 2020 and February 2021, who were evaluated using the QMGS. Each item in the QMGS was analyzed for distribution. Three methods of evaluating grip strength, grip strength decrement, maximum grip strength, and relative grip strength, were compared. The correlation between the QMG total score minus grip strength score, and three evaluating methods, was analyzed.

**Results:** The grip strength, swallowing, speech, diplopia, ptosis, and facial muscles items showed a clustered distribution. Most patients (94%) presented their maximum grip strength in the first four grip strength measurements. The QMG total score minus the grip strength score had a weak correlation with grip strength decrement (R grip *r* = 0.276; L grip *r* = 0.353, both *p* < 0.05) and moderate correlations with maximum grip strength (R grip *r* = −0.508; L grip *r* = −0.507; both *p* < 0.001) and relative grip strength (R grip *r* = −0.494; L grip *r* = −0.497, both *p* < 0.001).

**Conclusions:** This study suggested that partial items in the QMGS have low discriminative properties for Chinese populations and the maximum grip strength value is the better method to evaluate grip strength compared to the other two scoring methods. Based on the quartiles of maximum grip strength, we propose new scoring ranges for the grip strength items.

## Introduction

Myasthenia gravis is an autoimmune disease that affects the postsynaptic membrane at the neuromuscular junction, inducing weakness in different muscle groups (1). Various scales have been developed to evaluate muscle weakness comprehensively and to date, the quantitative myasthenia gravis (MG) score (QMGS) is one of the most commonly used.

The QMGS scale was first developed by Besinger et al. (2) in 1983 to measure the severity of symptoms. It consists of eight items, including five quantitative items (arm, leg, neck, grip, and vital capacity) and three qualitative items (facial muscles, chewing, and swallowing).

In 1987, Tindall et al. (3) added 2 ocular items (diplopia and ptosis), divided both arm and leg items into left and right scores, and adjusted the grip strength items from decrement after 10 maximal closures to a specific value.

In 1998, Barohn et al. (4) modified three items that could not be easily quantified in the prior scale (facial muscles, chewing, swallowing) and adjusted the vital capacity item from a specific value to a percentage of the predicted value.

In 2000, the Myasthenia Gravis Foundation of America (MGFA) recommended that a QMGS be applied in all prospective studies evaluating therapy for MG (5). Since then, the QMGS has been widely employed.

However, a Canadian study revealed that partial items of the QMGS held low discriminative properties given their marked floor effect (that is, a high proportion of patients with a score of 0 in these items) (6). Moreover, the scoring ranges of the QMGS were originally determined according to Western populations and there has been no research on the applicability of this scale in Chinese patients with MG. Among all items in the QMGS, the normal range of grip strength has regional variations (7). Chinese patients with MG may need a new scoring range for this item due to weaker grip strength observed in Asian populations than that in Western populations (7). In addition, the scoring ranges of the grip strength items were established over 30 years ago (3) and over time the reduction of manual labor may facilitate a gradual decrease in grip strength (6). Methods for evaluating grip strength include grip strength decrement (2), maximum grip strength (8), and relative grip strength (9). Presently, the QMGS uses the maximum grip strength for scoring, however, there has been no study comparing these three scoring items. Therefore, this study aimed to evaluate the discriminative properties of each QMGS item in Chinese populations, confirm whether the maximum grip strength is an acceptable assessment method, and explore new scoring ranges of the grip strength items.

## Methods

This was an investigator-initiated, single-center, prospective, cross-sectional, observational study. All experimental protocols were approved by the Ethics Committee of the First Affiliated Hospital of Guangzhou University of Chinese Medicine (NO. ZYYECK [2019] 055). The study conformed to the Declaration of Helsinki guidelines. Informed consent was obtained from all participants or a parent and/or legal guardian if participants were under 18 years of age.

This study included patients with MG enrolled from The First Affiliated Hospital of Guangzhou University of Chinese Medicine between September 2020 and February 2021. The QMGS evaluation was performed by a physician with experience in neuromuscular diseases, according to the MGFA version of the QMGS (5). All participants underwent an initial assessment without follow-up. Participants could continue their pre-assessment immunosuppressive and symptomatic treatments, and the administration time of pyridostigmine, a cholinesterase inhibitor, was recorded. A Jamar hydraulic grip dynamometer (Shanghai RuiShi Biological Technology Co., Ltd., Shanghai, China) and an electronic spirometer (Guangzhou Guanbo Medical Technology Co., Ltd., Guangzhou, China) were used. The grip strength of each hand was measured 10 times continuously and recorded in the form of a numerical value, rather than an item score. The maximum grip strength in 10 measurements was used to calculate the QMG total score. The grip strength decrement was calculated by the following formula: 1- (minimum grip strength/maximum grip strength). The relative grip strength was calculated by maximum grip strength/body mass index (9). The number of measurements on which the maximum grip strength occurred were recorded. Other items in the QMGS were recorded in the form of item scores.

The SPSS 25 software (IBM, Armonk NY, USA) was used for statistical analysis. We calculated the QMG total score of each subgroup in different classifications [sex, patient source, MGFA classification, subgroups based on the interval of pyridostigmine use, and subgroups based on serum antibodies and clinical feature (10)]. When the data were normally distributed, a two-sample *t*-test and one-way analysis of variance were used to compare the QMG total scores in the different subgroups. For multiple comparisons, a least significant difference *t*-test was used for homogeneous variance and Dunnett's *t*-test was used for heterogeneous variance. When the data were not normally distributed, a Wilcoxon rank-sum test and a Kruskal–Wallis test were used. For multiple comparisons, a Bonferroni *post hoc* correction was used. A *p*-value ≤ 0.05 was considered statistically significant. To evaluate discriminative properties, we analyzed the distribution of each item score and calculated the QMG total score of all the patients within each specific item score.

To evaluate the grip strength items, we compared three scoring methods: maximum grip strength, grip strength decrement, and relative grip strength. We calculated the quartile, mean, and standard deviation of the three scoring methods in both sexes. The correlation between grip strength and the QMG total score minus the grip strength score was analyzed using Pearson correlation. The absolute value of the correlation coefficient was classified and interpreted as follows: 0–0.09, negligible correlation; 0.1–0.39, weak correlation; 0.4–0.69, moderate correlation; 0.7–0.89, strong correlation; 0.9–1, very strong correlation (11).

## Results

A total of 106 patients participated in the study (Table 1). The mean age was 44.78 ± 15.98 years and 29 patients (27%) were male. The mean QMG total score in men was lower than that in women but was not statistically significant (*p* = 0.074). The mean QMG total scores in different patient source subgroups (*p* < 0.001), MGFA classification subgroups (*p* < 0.001), and interval of pyridostigmine use subgroups (*p* < 0.05) were all different. Patients who received pyridostigmine 4–5.9 h before the assessment had a high QMG total score; however, there was no significant difference compared to other interval subgroups except for the “not taking pyridostigmine” subgroup (*p* = 0.001).

**Table 1:** Demographic, clinical characteristics, and quantitative myasthenia gravis (MG) scores (QMGS) of the study patients.

**Characteristics**	**Mean ± SD or n**	**QMG score (mean ± SD)**
Age (year)	44.78 ± 15.98	
Height (cm)	160.59 ± 8.22	
Weight (kg)	57.09 ± 12.00	
Body Mass Index (kg/m^2^)	22.09 ± 3.87	
Sex (*n*)^*^		
Male Female	29 77	10.17 ± 6.57 12.66 ± 6.24
Patient source (*n*)^***^		
Inpatient Outpatient	42 64	16.36 ± 5.90 9.11 ± 4.94
MGFA classification (*n*)^***^^#^		
I IIa IIb IIIa IIIb IVa IVb V	3 55 17 14 13 4 0 0	4.00 ± 2.00 8.78 ± 3.94 10.29 ± 5.39 17.21 ± 3.60 20.69 ± 3.59 22.50 ± 3.87 / /
The interval of pyridostigmine (*n*)^**^^†^		
A: 0–1.9 h B: 2–3.9 h C: 4–5.9 h D: Over 6 h E: Not taking pyridostigmine	26 37 18 14 11	10.96 ± 5.15 12.38 ± 6.44 16.39 ± 7.22 11.07 ± 5.28 7.00 ± 4.73
Total (*N*)	106	11.98 ± 6.40

*The differences between QMG total scores in the different subgroups were analyzed. The sex and patient source were calculated by a two-sample t-test. The MGFA classification was calculated by one-way analysis of variance. The interval of pyridostigmine was calculated by a Kruskal–Wallis test*.

* *p > 0.05;*

** *p <0.05;*

*** *p <0.001*.

# *Further pairwise comparison showed that there was no significant difference between class IIb and class IIa and between class IIIb and IVa. The remaining pairs all showed significant differences (p <0.05)*.

† *Further pairwise comparison showed that the pair with a statistical difference was subgroup C and subgroup E (p = 0.001)*.

*MGFA, Myasthenia Gravis Foundation of America; QMG, quantitative myasthenia gravis*.

**Figure 1:**
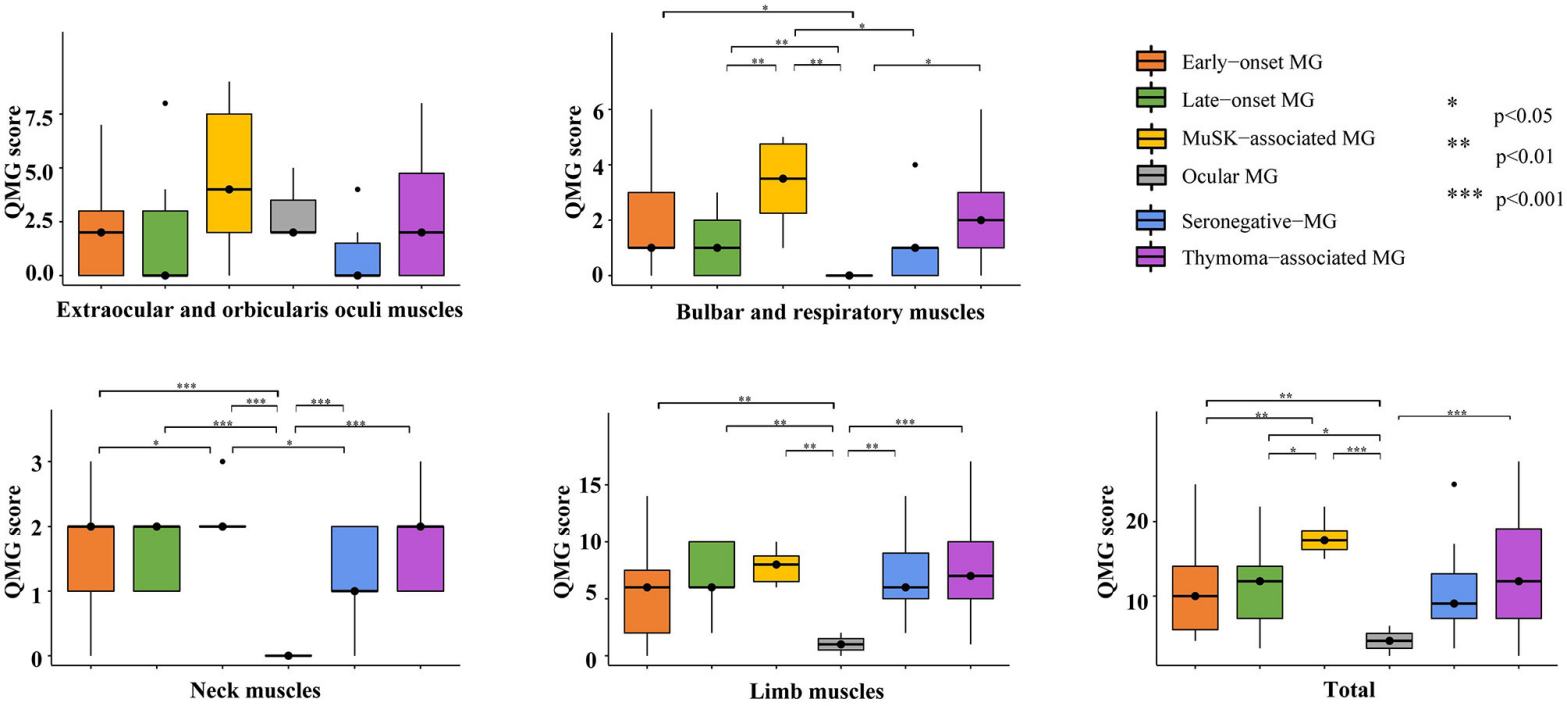
Multiple comparisons of each muscle group in the serum antibodies and clinical features subgroups. MuSK-associated MG had high scores for bulbar, respiratory, and neck muscles and high QMG total scores. MG, myasthenia gravis; QMG, quantitative myasthenia gravis; MuSK, muscle-specific kinase.

Table 2 and Figure 1 depict the QMG scores of different muscle groups in serum antibodies and clinical features subgroups. There was no significant difference in extraocular and orbicularis oculi muscles of any of the subgroups or the limb muscles of all generalized-MG subgroups. A muscle-specific kinase (MuSK)-associated MG had a higher score for bulbar and respiratory muscles (compared to late-onset MG, seronegative-MG, and ocular MG) and neck muscles (compared to early-onset MG, seronegative-MG, and ocular MG) and a higher total score (compared to early-onset MG, late-onset MG, and ocular MG).

**Table 2:** QMG scores of different muscle groups in the serum antibody and clinical feature subgroups.

**Subgroups based on serum antibodies and clinical features**	***N***	**QMG total score**	**Extraocular and orbicularis oculi muscles**	**Bulbar and respiratory muscles**	**Neck muscles**	**Limb muscles**
Early-onset MG	39	10.90 ± 5.72	1.95 ± 1.82	1.90 ± 1.64	1.41 ± 0.75	5.64 ± 3.57
Late-onset MG	13	11.08± 5.54	1.62 ± 2.47	1.08 ± 1.04	1.54 ± 0.52	6.85 ± 2.70
MuSK-associated MG	6	17.83 ± 2.48	4.50 ± 3.67	3.33 ± 1.63	2.17 ± 0.48	7.83 ± 1.60
Ocular MG	3	4.00 ± 2.00	3.00 ± 1.73	0.00 ± 0.00	0.00 ± 0.00	1.00 ± 1.00^*^
Seronegative-MG	11	10.72 ± 6.24	0.82 ± 1.33	1.45 ± 2.16	1.36 ± 0.67	7.09 ± 3.83
Thymoma-associated MG	34	13.64 ± 7.16	2.59 ± 2.63	2.15 ± 1.93	1.65 ± 0.65	7.26 ± 3.70

*QMGS items were divided into different muscle groups as follows: Extraocular and orbicularis oculi muscles: diplopia, ptosis, facial muscles (lid closure) items; Bulbar and respiratory muscles: swallowing, speech, vital capacity items; Neck muscles: head lifted item; Limb muscles: left and right arm outstretched, left and right-hand grip, left and right leg outstretched items.*

* *There were two ocular patients with MG who had scores in the grip strength items*.

*MG, myasthenia gravis; QMG, quantitative myasthenia gravis; MuSK, muscle-specific kinase*.

**Figure 2:**
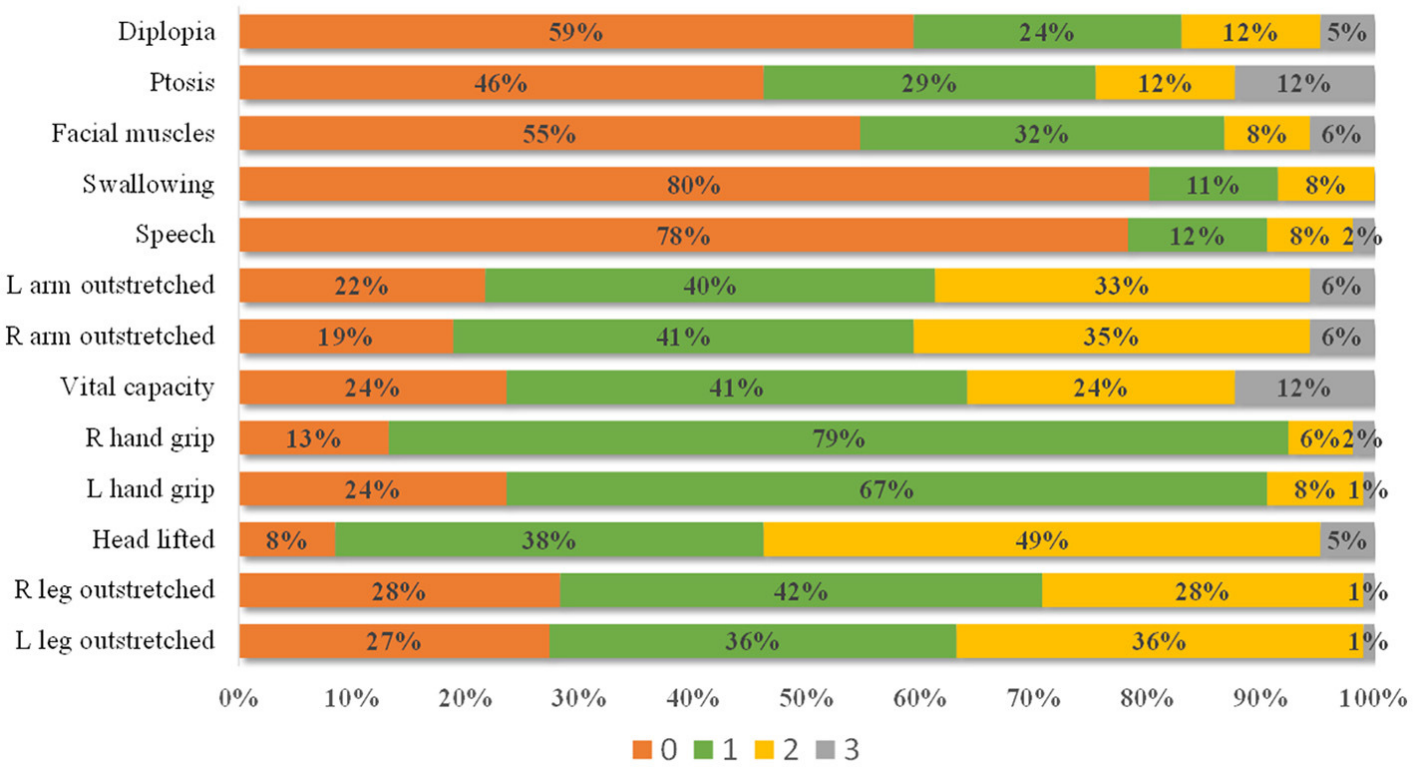
The distribution of each item score. Most patients present with a 0 score for diplopia, ptosis, facial muscles, swallowing, and speech items, 1 score for left and right grip strength, and 2 scores for head lifted.

The distribution of each item score is shown in Figure 2. Most patients received a score of 1 for grip strength (79% for the right hand and 67% for the left hand) and a score of 0 for swallowing (80%) and speech (78%). A score of 0 scores for diplopia (59%), ptosis (46%), and facial muscles (55%), and a score of 2 for head lifted (49%) also showed a clustered distribution. In general, patients with a high item score had a high QMG total score (Table 3), indicating that the setting of items conforms to MG severity.

**Table 3:** The QMG total score of all the patients in each specific item score^†^.

**Item**	**QMG total score in each item score segment [mean** **±** **SD, (*****n*****)]**				**Pairwise comparison**
	**0**	**1**	**2**	**3**	
Diplopia	9.73 ± 5.54 (63)	12.72 ± 4.60 (25)	19.85 ± 6.26 (13)	16.20 ± 6.94 (5)	02^***^
Ptosis	9.00 ± 5.22 (49)	12.39 ± 6.02 (31)	17.77 ± 5.53 (13)	16.46 ± 6.72 (13)	02**, 03^***^
Facial muscles	8.79 ± 4.19 (58)	13.85 ± 6.15 (34)	19.00 ± 5.37 (8)	22.83 ± 2.79 (6)	01**, 02^***^, 03^***^
Swallowing	10.71 ± 5.72 (82)	17.17 ± 6.01 (12)	19.33 ± 3.71 (9)	/ (0)	01**, 02^***^
Speech	10.83 ± 5.51 (80)	14.92 ± 6.59 (13)	20.88 ± 4.16 (8)	15.50 ± 13.44 (2)	02^***^
R arm outstretched	6.00 ± 2.60 (20)	9.95 ± 4.82 (42)	16.63 ± 4.09 (35)	23.00 ± 3.85 (6)	02^***^, 03^***^, 12^***^, 13^***^
L arm outstretched	6.35 ± 2.64 (17)	8.86 ± 4.06 (43)	17.16 ± 4.18 (37)	22.33 ± 3.78 (6)	02^***^, 03^***^, 12^***^, 13^***^
Vital capacity	6.68 ± 3.71 (22)	10.26 ± 4.55 (43)	16.12 ± 4.79 (25)	20.54 ± 4.67 (13)	02^***^, 03^***^, 12**, 13^***^
R hand grip	6.08 ± 3.88 (13)	12.45 ± 5.80 (82)	18.17 ± 5.46 (6)	24.50 ± 4.95 (2)	01**, 02^***^, 03**
L hand grip	8.04 ± 4.88 (23)	12.50 ± 5.84 (70)	18.89 ± 4.49 (9)	28.00 ± 0.00 (1)	01^*^, 02^***^, 12^*^
Head lifted	5.00 ± 1.67 (6)	7.20 ± 3.11 (40)	15.92 ± 4.57 (52)	22.40 ± 4.51 (5)	02^***^, 03^***^, 12^***^, 13^***^
R leg outstretched	5.70 ± 2.44 (27)	11.69 ± 4.04 (45)	18.57 ± 5.16 (30)	21.00 ± 0.00 (1)	01**, 02^***^, 12^***^
L leg outstretched	5.92 ± 2.46 (26)	11.11 ± 4.42 (38)	17.39 ± 5.31 (38)	21.00 ± 0.00 (1)	01**, 02^***^, 12^***^

*A Kruskal–Wallis test was used to analyze the QMG total score in different score segments of each item. All items have a p-value <0.001. The paired groups with significant differences are shown in the last column (Each subgroup is represented by the item score, for example, “01” means the 0 score subgroup vs. the 1 score subgroup).*

† *Expect for diplopia, ptosis, and facial muscles items, three ocular MG data had been excluded in the remaining items*.

* p <0.05; ^*^p <0.01;

*** *p <0.001.*

*QMG, quantitative myasthenia gravis; MG, myasthenia gravis*.

**Figure 3:**
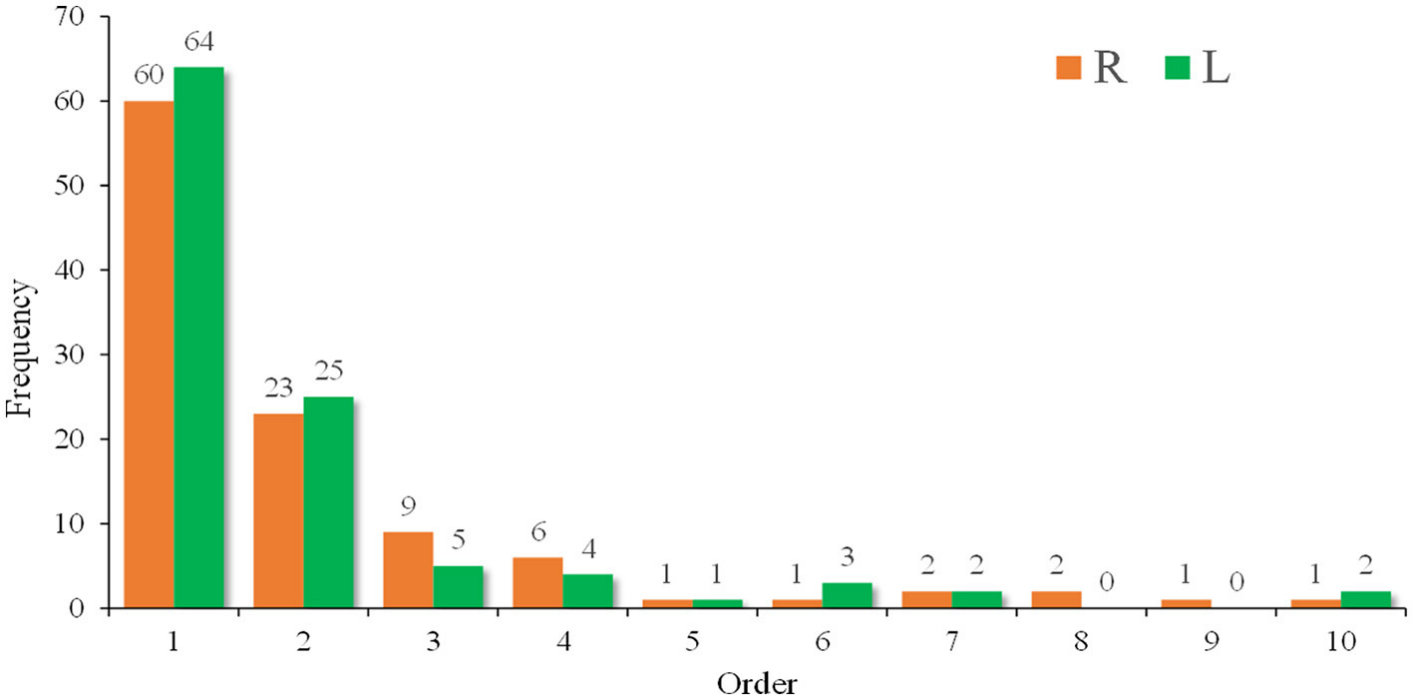
A frequency diagram of the order in which the maximum grip strength occurred. Most patients (94%) presented their maximum grip strength (both left and right) in the first four measurements.

The order in which the maximum grip strength occurred is shown in Figure 3. Most patients (94%) presented their maximum grip strength (both left and right hands) in the first four measurements. Table 4 shows the performance of the three methods of evaluating grip strength. The maximum grip strength and relative grip strength had significant differences between men and women (both *p* < 0.001) while grip strength decrement did not (*p* > 0.05). The QMG total score minus the grip strength score had a weak correlation with grip strength decrement (R grip *r* = 0.276; L grip *r* = 0.353; both *p* < 0.05) and was moderately correlated with maximum grip strength (R grip *r* = −0.508; L grip *r* = −0.507; both *p* < 0.001) and relative grip strength (R grip *r* = −0.494; L grip *r* = −0.497; both *p* < 0.001).

**Table 4:** Performance of 3 methods on evaluating grip strength^†^.

	**R grip strength decrement**	**L grip strength decrement**	**R maximum grip strength**	**L maximum grip strength**	**R relative grip strength**	**L relative grip strength**
Mean ± SD^*^						
Male: Female:	36.43 ± 12.28 37.75 ± 17.08	36.16 ± 14.01 37.22 ± 15.45	32.55 ± 13.51 kg 20.06 ± 6.76 kg	30.96 ± 12.56 kg 18.77 ± 6.18 kg	1.44 ± 0.59 0.93 ± 0.35	1.36 ± 0.52 0.88 ± 0.34
P_25_ Male: Female:	45.14 48.29	39.61 47.01	21.80 kg 15.30 kg	22.90 kg 14.20 kg	0.92 0.71	1.06 0.64
P_50_ Male: Female:	36.12 33.96	33.74 35.83	34.50 kg 19.20 kg	32.30 kg 18.50 kg	1.51 0.88	1.47 0.87
P_75_ Male: Female:	27.91 25.38	27.84 24.46	40.50 kg 25.45 kg	39.30 kg 23.35 kg	1.86 1.17	1.73 1.10
correlation coefficient	0.276	0.353	−0.508	−0.507	−0.494	−0.497
*p*-value	0.005	<0.001	<0.001	<0.001	<0.001	<0.001

*Three ocular MG data had been excluded*.

* *There were significant differences in maximum grip strength and relative grip strength between males and females (All p-values <0.001). There was no significant difference in grip strength decrement between males and females (R grip p = 0.713; L grip p = 0.754)*.

## Discussion

It is a dilemma whether to take cholinesterase inhibitors before the QMGS assessment. Pyridostigmine before an assessment can alleviate symptoms and reduce the QMG total score, which creates an observation bias. In contrast, the discontinuation of pyridostigmine before the assessment requires good adherence and may have adverse effects on the patients. When the predecessor of the QMGS was first developed in 1983, Besinger stipulated that the scale should be used within 3 h of the last pyridostigmine dose, during the morning (2). Thereafter, most studies have not specified whether taking pyridostigmine before the assessment is advisable. In this study, there was no time limitation on pyridostigmine administration. We found that patients who took pyridostigmine 4–5.9 h before the assessment had a high QMGS (this may be because the drug effect had not degraded in patients with short intervals, while patients with long intervals had a mild disease condition); however, due to insufficient sample size in each subgroup, the differences were not statistically significant.

During the assessment, a portion of the patients gradually became ptotic when assessing diplopia, which interfered with the evaluation of the ptosis item. Our solution was to assess this item at the last after ptosis was improved. We speculate that it would also be a solution to assess these two ocular items together by gazing at the upper outer quadrant; however further studies are required for confirmation. Moreover, the diplopia item is subjective as it depends on the patient's report. Some patients complained of blurred vision rather than diplopia throughout the assessment.

The speech item is the most difficult item to assess accurately. Different languages and dialects have different pronunciation characteristics and some numbers are likely to present dysarthria (for example, “three” in Mandarin Chinese). Moreover, because dysarthria usually presents gradually and researchers do not know what the patients' accents were when they were healthy, the judgment tends to be subjective.

The vital capacity item also has its shortcomings. There are racial and ethnic variations in vital capacity and the population-specific standards are recommended (12). However, these standards have not been used in most current spirometers in China. The process of table-lookup or calculation is also inconvenient. In this study, the predicted value of vital capacity was calculated by the spirometers automatically. We found that most patients' vital capacity values were not in accordance with those previously reported (6).

The result of maximum grip strength in this study is similar to that reported in another Chinese MG study (8). Since 94% of patients presented their maximum grip strength in the first four measurements (Figure 3), we suggest that the grip strength item should be measured four times to obtain the maximum value. In 1987, Tindall adjusted the grip strength item from decrement after 10 maximal closures to a specific value; however, he did not explain the reason (3). In our study, grip strength decrement had a weak correlation with the QMG total score minus the grip strength score (Table 4), indicating that it may not accurately reflect symptom severity. Both relative grip strength and maximum grip strength showed sex differences (*p* < 0.001) and required setting different scoring ranges for males and females. However, when relative grip strength was calculated, it performed similarly to the maximum grip strength (both with a correlation coefficient of ~-0.5). Given these results, we considered the maximum grip strength to be an acceptable method. As shown in Figure 2, the grip strength items in the QMGS could not distinguish the weakness among patients well, and a majority of patients had both left and right-hand grip strength within the range of 1. Therefore, the grip strength scoring range might need to be revised for even distribution. In Table 4, the quartiles divide the data into four equal parts, which can correspond to the four segments of the QMG scoring range. However, those quartiles are not integers, which would be inconvenient to practical applications. In addition, grip strength increases to a peak in early adult life are maintained through to midlife and then declines (13). Still, there has been no study evaluating grip strength in elderly patients with MG in China. Given the inherent decline in grip strength, it seems acceptable to ignore the decline in grip strength in the elderly when setting the scoring range. Therefore, according to the quartiles of maximum grip strength and for simplicity, we propose that for Chinese populations, the scoring ranges of both left and right grip strength items in the QMGS could be set as follows: 3 scores, 0–20 kg for men and 0–15 kg for women; 2 scores, 20–30 kg for men and 15–20 kg for women; 1 score, 30–40 kg for men and 20–25 kg for women; 0 score, ≥40 kg for men and ≥25 kg for women. The above ranges are semi-open closed intervals, the lower bound is closed while the upper bound is open. According to the original QMG scoring ranges or the new ranges, the mean score of the sum of left and right grip strength items was 1.83 ± 1.02 and 2.97 ± 2.12, respectively. When applying the new ranges, the variable coefficient increased from 0.56 to 0.71, indicating a discrete distribution.

In this study, we included a few ocular MG and MuSK-associated patients with MG, and there were no low-density lipoprotein receptor-related protein 4 (LRP4)-associated patients with MG for its commercial tests had not been universal. Therefore, it should be circumspect to interpret the result of the QMGS in serum antibodies and clinical features subgroups. In addition, few patients with severe MG (MGFA IV and V) were included in this study, which could create a bias.

Some clinical trials have found that the QMGS scale was less sensitive than other scales like the myasthenia gravis activities of daily living profile (MG-ADL) (14–16). This may be attributed to the following reasons: the QMGS mainly assesses generalized weakness while the MG-ADL mainly assesses oculobulbar weakness (17); the QMGS is a point-in-time measure while the MG-ADL uses patients' recollections from the previous week (18). We found that some QMGS item scoring ranges were too broad. Many patients got the same score in diplopia, ptosis, facial muscles, swallowing, speech, grip strength, and head lifting items, indicating that these items did not show good discriminative properties. This may also be one of the reasons for the low sensitivity of the QMGS. Some researchers considered using QMGS items as continuous variables which may improve the sensitivity of the scale (14). However, this would complicate the data analysis as a patient may simultaneously present with both improved and worsened items. To improve the discriminative property of the QMGS, new item scoring ranges may be needed to be established.

Therefore, to further modify the QMGS, we are conducting another study. We assess the QMGS in patients with MG and healthy, sex-, age-, height-, and weight-matched groups, and record the items as numerical values. A receiver operating characteristic curve will be used to set the 0 scoring range of an item based on the two groups' data. The remaining patients with MG who cannot receive a 0 score will be divided into three subgroups by tri-sectional quantiles of assessed data. These three subgroups will represent the 1, 2, 3 scoring range of the item, respectively. Through this procedure, the QMG scoring distribution would be even and the discriminative property of the scale would be improved.

## Conclusions

In this observational study, we included 106 Chinese patients with MG and evaluated the QMGS. We found that the grip strength, swallowing, speech, diplopia, ptosis, and facial muscles items held low discriminative properties. To obtain the maximum grip strength, it should be measured four times. The maximum grip strength assessment was the more effective method compared with grip strength decrement and relative grip strength. Given these findings, we proposed new scoring ranges for this item.

## Data Availability Statement

The raw data supporting the conclusions of this article will be made available by the authors, without undue reservation.

## Ethics Statement

The studies involving human participants were reviewed and approved by First Affiliated Hospital of Guangzhou University of Chinese Medicine. Written informed consent to participate in this study was provided by the participants' legal guardian/next of kin.

## Author Contributions

QJ, JLi, SW, and XY contributed to the study conception and design, acquisition of funding, and interpretation of the data. JLi, SW, LH, BL, and JLu evaluated the participants using the QMGS. JLi and SL were responsible for statistical analysis and drafting of the manuscript. All authors read and approved the final manuscript.

## Funding

This study was supported by the National Natural Science Foundation of China (8190413), the Youth Innovation Scientific Research Project of The First Affiliated Hospital of GZUCM (2019QN28), and the Scientific Research Team Training Project Of GZUCM (2019KYTD101). None of the funding sources took any part in the study design, data collection, and analysis, interpretation of data, or in the writing of the manuscript.

## Conflict of Interest

The authors declare that the research was conducted in the absence of any commercial or financial relationships that could be construed as a potential conflict of interest.

## Publisher's Note

All claims expressed in this article are solely those of the authors and do not necessarily represent those of their affiliated organizations, or those of the publisher, the editors and the reviewers. Any product that may be evaluated in this article, or claim that may be made by its manufacturer, is not guaranteed or endorsed by the publisher.
